# The "Art of Rising" in Physic

**Published:** 1861-04

**Authors:** 


### Art. XIV.—THE "ART OF RISING" IN PHYSIC.

The *Medical Directory* for Great Britain, now for the first time published in a single volume, and containing the names of more than 16,000 practitioners in various departments of the healing art, can hardly fail to suggest important questions with regard to the sources of demand for so much talent, and with regard to the means by which individual members of the profession contrive to raise themselves above the respectable mediocrity of the great mass of their brethren. The pass-lists of the examining bodies show that the yearly average of additions to the licentiates in physic and surgery considerably exceeds 1000, and hence, to this number of young men at least, the art of rising in physic embraces questions upon the solution of which the conduct of their lives must turn. And not of their own lives only; for their relations with the public are of a nature to cause the influence of their example to be both widely spread and deeply felt; so that the doctor, in no small degree, reacts upon and moulds the characters of his patients.

Presupposing the possession of a sufficient income, either from professional or private sources, to remove any anxiety about daily wants, it would seem, *a priori*, that the medical profession, of all possible callings, is the one most calculated to develope every intellectual and moral excellence to which man, in this life, can attain. Removed from the larger ambitions of the world, from the jobberies of political craft, and from the ostentations of commercial prosperity,—familiar with the summons of the dread visitant that dissipates the visions of human grandeur, and prevents the anticipated triumphs of human skill,—standing upon the threshold of sciences that shadow forth, even as we see them in their veriest rudiments, something of the power and the wisdom of Him in whose fiat they have had their origin,—placed in a path of duty that harmonizes, in outward deed at least, the love of self with the love of the neighbour, and makes the benefit of others the surest passport to success,—the healing art might be expected to combine the advantages of a life of practical benevolence with those of a life of philosophic contemplation ; and might afford to its followers at once the benefits of activity, and the dignity of repose.

And if we glance, either retrospectively or around us, at the worthies of our craft, we shall find that many among them have displayed, and that many still display, a perfect consciousness of the elevation of their calling. Not only in the daily lives of men whose scientific eminence has made us acquainted with the most trivial details of their conduct, but also in the general tone of thought which prevails among the public with regard to the most respected practitioners of any given locality, this consciousness is practically shown. That there should be numerous exceptions to the rule is a twofold necessity, depending both upon the essentially intrinsic character of human error, and very greatly also, upon external circumstances.

For, in the anxieties of life, and in some measure from the circumstance that the licensing bodies of the profession have opened a door cheaply for the admission of students whose special education has been meagre, and whose general education has been positively *nil*, added to this, that the possession of a qualification has usually enabled a single man to obtain an immediate pittance as an assistant or a parish doctor, numbers of persons have become legally qualified to practise without possessing either the pecuniary means or the mental culture necessary in order to enable them to wait patiently for that moderate portion of success which is the invariable reward of the deserving. Such men as these, often with considerable talent and energy, but usually throughout their lives conspicuous rather by their activity than their weight, create and maintain a turbid and eddying backwater by the side of the steady current of professional opinion. If they eventually reach high places, their examples tend to confusion of thought about the first principles of ethics ; and frequently beguile the inexperienced into a belief in the existence of an art of rising, to be studied separately from the art of healing. They have induced some, it may be feared, to give an exclusive attention to the former ; and to neglect their proper studies in quest of some imaginary, often underhand, means of "getting on ;" in accordance with the vulgar superstition that so often attributes the success of the successful man to his weakness, which can be imitated, rather than to his strength, which cannot.

The most marked illustration of this principle that our own day has furnished was the prevalence of what may be termed the "Abernethy manner," during, and subsequent to, the latter part of the career of the great man whose outermost shell of peculiarity was thus feebly travestied. Full of years and honours, it was the practice of John Abernethy to be plain-spoken—to let his strong common-sense assert itself against empty pretension, and his wit to flash its scorn of arrogance or affectation. Having acquired, by careful study of innumerable details, and by a vast and varied experience, the power to decide rapidly upon most professional questions, and having his day by far too short for his daily labours, it was his practice to save himself, and to forbid his patients, any indulgence in the loquacity that loves to babble of empty trivialities. And so, the youthful aspirant for professional success, forgetting that this manner was greatly the result of circumstances—forgetting that it was, in many instances of its display, an unsightly blemish upon a most benevolent and noble character—forgetting that manner, in order to be impressive, must always be original, the natural outgrowth of the mind—and too often forgetting that no man has a right to be decided, unless his decision rest upon hard study and wide experience—forgetting all these things, has sometimes brought a fancied imitation of his great teacher to the bedside of the humble and the poor, has checked, with cowardly bearishness, or coarse *brusquerie*, the timid relation of symptoms often of grave import, and has justified the bitter lines of the satirist, "Nature's sternest painter, yet the best:"—

> But soon a loud and hasty summons calls,
> Shakes the thin roof, and echoes round the walls;
> Anon, a figure enters, quaintly neat,
> All pride and business, bustle and conceit,
> With looks unaltered by these scenes of woe,
> With speed that, entering, speaks his haste to go;
> He bids the gazing throng around him fly,
> And carries fate and physic in his eye;
> A potent quack, long versed in human ills,
> Who first insults the victim whom he kills;
> Whose murderous hand a drowsy bench protect,
> And whose most tender mercy is neglect.
> Paid by the parish for attendance here,
> He wears contempt upon his sapient sneer;
> In haste he seeks the bed where misery lies,
> Impatience marked in his averted eyes;
> And, some habitual queries hurried o'er,
> Without reply, he rushes to the door.

There are not wanting, however, persons with whom this style of demeanour is almost necessary, nor places where it may be used with effect. Among the members of small communities, always prone to magnify that which appertains to their locality, always certain, as an adjunct to their own consequence, to have the best doctor and the cleverest lawyer in the kingdom, and especially in communities recently enriched by commerce and only dimly enlightened by education, arrogant self-assumption is very frequently successful. Pretenders whom Abernethy would have crushed under a sarcasm, flourish weapons from his armoury in the faces of the public. We lately heard a description of a certain Dr. Bouncer, thus situated, who was called in consultation to the abode of opulent Mr. Jones. Following the general practitioner in attendance into the drawing-room where the ladies of the family were assembled, the doctor rapidly divested himself of his great coat, threw it past the astonished head of the mistress of the house, to alight upon a distant chair, arranged himself in a recumbent posture upon the most comfortable-looking sofa in the room, and then first breaking the silence that had ensued upon his entrance, exclaimed, with an authoritative wave of his hand, "*Mrs. and Miss Jones, be good enough to retire.*" "Sir," said Mr. Dosey, the general practitioner, in recounting the matter on the same evening, "Sir, the way little Bouncer goes to work takes my breath away. Those ladies hurried out of the room as if it did not belong to them; and there was he, who had never seen them before, with his muddy boots on the best sofa, asking questions of me about the case, and as cool as a cucumber." Poor Dosey was so much impressed, in fact, that he insulted his best patient the very next morning; and, sad moral, lost him by doing so. Bouncer, however, knew his ground, and was secure. Mrs. Jones was a humble captive at his chariot wheels, and shortly after sang his praises to a shrewd old Scotchman. "His manner is so imposing," said the lady. "Eh, y're aboot reet there," was the dry response; "he's a vara great impostor!"

Our readers will be familiar, too, with the other extremity of the scale, the courteous and supple doctor, whose face is creased into smiles, whose speech is a succession of acquiescences, and whose patients suffer under maladies of the most profoundly curious and instructive character, causing him to feel the "deepest interest" in their respective cases, and the greatest respect for their own opinions about symptoms and their causes. Of such a man wrote Cowper, in the familiar lines:—

> *Dubius* is such a scrupulous good man—
> Yes—you may catch him tripping if you can.
> He would not, with a peremptory tone,
> Assert the nose upon his face his own;

With hesitation admirably slow,
He humbly hopes—presumes—it may be so.

"I assure you, my dear Miss B——," we once heard a specimen of this class say to a rich old maid, "that I sat yesterday for half an hour, with my pen suspended, pondering whether I should decide upon the sixth or the seventh of a grain as the dose best suited to your state." It is frequently a matter for grave consideration among young practitioners, to which of these opposing schools they shall attach themselves at the outset of their career; and it often happens that they make the worst possible choice. Men of spirit, who might successfully carry off a tone of great decision, cultivate an artificial blandness; and men with chicken hearts, accustomed to be influenced by the pretensions or the bluster of others, carry their bluster to the side of the sick bed. Artificial blandness, moreover, is not uncommonly aided by artificial piety. We once travelled from London to the north with a young surgeon who had determined to establish himself at some town on the route, and who had made the most minute inquiries as to the religious creed which in each place it might be most to his advantage to profess. He related the results of his investigations with surpassing *naïveté*. "If I come here," he said, as the train slackened speed near to one of the stations, "I shall have to be a Methodist. They are a very strong body in the place; and of the men now in practice, four are Churchmen, one is a Romanist, and one a Baptist. Now at E——, ten miles further on, I should have to be a Catholic." It is a melancholy fact, that, in the midland and northern districts of England, and especially in the large towns, the professional connexions of inferior practitioners depend very greatly upon their respective chapels; and where there is the obvious prospect of temporal rewards attaching to certain professions, it is impossible not to suspect that such professions, when very loudly and fiercely made, may arise from motives having at least some admixture of worldliness; that they may, in a word, be nothing better than experiments in the art of rising.

Next to artificial piety, we should be disposed to think that sham science is regarded as the most effectual stepping-stone on the road of social and professional progress. The possession of a little philosophical apparatus has been the basis of reputation to many a Sir Frizzle Pumpkin in the medical world; and there are few things more charming than the babble of a certain class of patients about the attainments that the possession of such apparatus is supposed to indicate. A faith as simple as profound has ere now been pinned upon a Leyden jar or an air-pump: not to speak of a microscope, the owner of which might not be able to tell a cancer-cell from an air-bubble. Even the aquarium has not been without its uses; and we know of one doctor who has most energetically employed that elegant means of studying the manners and customs of subaqueous life, as a wedge by which to insinuate himself into families. Perfectly skilled in all the details of aquarium management, he sees one of the crystal cases through the window of a house by which he has to pass, but the residents in which are unknown to him. To pull up—to alight—to call—to introduce himself to the ladies—to apologize a hundred times for his intrusion—to explain that he thought he had caught a glimpse in the very beautiful aquarium in the window, of a fine specimen of the *Ægra inepta*, which he himself had often tried in vain to cultivate—to discover his singular error—to beg that he might be excused on the ground of his enthusiasm—to depart—to call again—to offer some diving spiders—to call a third time to inquire how the spiders were prospering, &c. &c. Such was the routine that has laid the foundation of at least one prosperous practice.

Of such shifts and schemes as these, and of the thousand and one tricks of the Bob Sawyer school that are daily practised, indefensible as they are on many grounds, it is hard to speak in the terms that perhaps they strictly merit. The persons who use such means of obtaining notoriety are often necessitous, and think, perhaps justly, that if they could obtain patients, they could treat them as well as their neighbours. The public are, and must remain, utterly unable to appreciate the gradations of professional ability. They argue from the general to the particular, and think that if a man be what they call "clever," he must be clever in professional matters;—a sufficiently just inference, if only he have paid the necessary degree of attention to them. This, every doctor is of course supposed to have done, unless his mind and thoughts are notoriously given to other subjects. But the popular tests for cleverness reach only to the discovery of tact and *savoir faire*. If, therefore, a man can see his own proper line and keep to it, being bombastic if his gifts incline that way, bland and conciliating if of a weak and humble spirit; if he can accommodate himself to the vices or the prejudices of the class into which he is thrown; if he can ride triumphant upon the latest wave of quackery or popular delusion, can call spirits from the vasty deep, or deliver an oracular opinion concerning the verity of Darwinism; each or all of these accomplishments will find its use in life, and will stamp the basest alloy with currency in the estimation of some section of the public. Through such dubious channels he may often procure his earliest clients, and may thus obtain an opportunity of building up a reputation of an unobjectionable character.

There is, however, an art of rising by means unmixedly base—

by artifices unmixedly contemptible. There are those who seek their elevation in the downfall of others, and who are constantly endeavouring to supplant their brethren. In London, for instance, it would not be difficult to name surgeons or physicians who, by some singular coincidence, invariably supersede the luckless practitioner who may call them in. Every particular case, of course, admits of great palliation, or even of entire explanation to every unprejudiced mind; but the point of the matter is, that in the practice of certain individuals the cases are so numerous and so similar.

Mrs. Bunny, in her third labour, was the subject of a shoulder presentation; and her medical attendant, anxious to diminish the responsibility resting upon him, suggested the co-operation of a distinguished accoucheur, who came, saw, turned, took his fee, and departed. A twelvemonth later a relative of Mrs. Bunny consulted the "great deliverer" at his house; and, having waited an hour and a half for her interview, in the company of other patients, the *Times* of yesterday, and a liberal supply of envelopes that had contained telegraphic messages, naturally exercised all her ingenuity to lengthen the brief period of time that the man of skill was ready to accord in exchange for her guinea. At her wits'-end, and almost at the door of the consulting-room, she exclaimed, as if by inspiration, "You remember my cousin Mrs. Bunny, Dr. Victory, and what a terrible time she had until you came to her assistance? Well, she is expecting again!" The fine countenance of the doctor became overspread with sudden consternation; and, falling a step backwards, he exclaimed, "Is it possible?" "Dear me!" responded the lady, "you do not anticipate any danger?" "Oh no, no, no," said the learned man, recovering his self-possession, "not at all—not at all, my dear madam—not at all. Mrs. Bunny will be excellently cared for by my friend Stick-in-the-mud; and, if anything perilous should happen, why—I can soon be summoned." Off went the cousin to poor Mrs. Bunny, and assured her that Dr. Victory was quite alarmed about her. "He seemed really shocked to hear of your state, and I am sure he expects something dreadful to happen, although he would not say so. But he completely betrayed himself at first; and I am sure, dear, if I were you, I would not trust to that Mr. Stick-in-the-mud. You had much better engage Dr. Victory at the first." And so it befell.

There is a prospect, however, of a future for the medical profession, in which the various shifts and arts to which we have referred will fall gradually into desuetude. They spring, we believe, mainly from ingenuity sharpened by need, and not controlled by education. It has been the object of the medical examining boards to throw open their portals as widely as possible, to make a bridge for every druggist's assistant and every needy student, to render it possible to fulfil the letter of a curriculum of study, while really engaged in other and laborious duties, and to admit within their pale men whose reasoning powers had never been properly exercised, and whose special knowledge was of the most imperfect kind. A new era now happily dawns. The Medical Council requires some evidence of a liberal education to be given before entering upon special studies at all, and the result will be that large classes from which the profession has been recruited in former times will for the future be excluded from its ranks. We congratulate ourselves much—we congratulate the public more. We look forward to a time when the profession of medicine, as of the Church or the Bar, will comprise only men of education and of knowledge, men who are aware that their duties in life are twofold, to *know* and to *do*. In these simple words the whole "art of rising" ought to be expressed; and is expressed, even now, excepting for those who have entered upon the battle of life with means inadequate to their requirements. For all others, to know their work thoroughly, and to do it well, constitute a sure passport to success; that is to say, to universal respect and moderate competence. Even in competition with the most unscrupulous, the essentials we have indicated must eventually secure the public confidence; and the honest doctor, who has studied his work thoroughly, and who does it well, need have but few anxieties with regard to his professional career. It is his privilege to stand aloof from, and above, party cabals and quarrels, to shun tricks and contrivances, whether for his own exaltation or for the downfall of his neighbour, and neither to damn with faint praise nor to criticize with envious censure those who may be placed in a position of rivalry to himself. We know many such men even now, honest, skilful, upright, laborious, sincere, carrying charity into their daily path of duty, using for good the manifold opportunities of their vocation, and reaping a rich reward in the approval of their own consciences, and the esteem of their fellow-citizens.

